# Relieving or Aggravating? The Longitudinal Moderating Role of Challenge and Hindrance Academic Stressors on the Relationship Between Leisure-Time Physical Activity and Context-Specific Anxiety Symptoms Among University Faculty

**DOI:** 10.3390/bs16050718

**Published:** 2026-05-07

**Authors:** Haozhen Li, Mengyu Shi, Qiuhan Zhu

**Affiliations:** 1 School of Kinesiology and Physical Education, Zhengzhou University, Zhengzhou 450001, China; lihaozhen01@zzu.edu.cn; 2 Physical Education and Training Institute, Capital University of Physical Education and Sports, Beijing 102600, China; shimengyu@cupes.edu.cn

**Keywords:** university faculty, leisure-time physical activity, emotional exhaustion, context-specific anxiety symptoms, challenge–hindrance academic stressors, cross-lagged panel model

## Abstract

**Objective:** Against the backdrop of high-intensity academic evaluations, this study examines the longitudinal associations linking leisure-time physical activity (PA) with context-specific anxiety symptoms among university faculty. It further examines the cross-lagged indirect role of emotional exhaustion (EE) and the contrasting moderating effects of challenge and hindrance academic stressors. **Methods:** A three-wave cross-lagged panel design spanning six months was employed to track 356 faculty members from five universities in China. Validated scales were utilized to measure leisure-time PA, challenge/hindrance academic stressors, EE, and context-specific anxiety symptoms across three critical academic periods (T1, T2, and T3). Data were analyzed using Mplus 8.3 for longitudinal measurement invariance, cross-lagged path modeling, and Bootstrap mediation and moderation tests. **Results:** After controlling for baseline autoregressive effects, T1 leisure-time PA significantly and negatively predicted T2 EE (β = −0.16, *p* < 0.01), which in turn positively predicted T3 context-specific anxiety symptoms (β = 0.31, *p* < 0.001). The longitudinal indirect pathway linking T1 PA to T3 context-specific anxiety symptoms through T2 EE was statistically significant (95% CI [−0.088, −0.017]). Furthermore, the two types of stressors exhibited contrasting moderating effects on the PA-EE relationship. High challenge stressors strengthened the negative association between PA and subsequent EE (Simple Slope = −0.32, *p* < 0.001). Conversely, high hindrance stressors were associated with a reversal of the slope, wherein higher PA involvement was associated with higher subsequent EE (Simple Slope = 0.12, *p* < 0.05), a pattern consistent with what has been termed an “exercise paradox.” **Conclusions:** In this three-wave observational study, regular leisure-time PA was longitudinally associated with, but did not demonstrably cause, lower subsequent context-specific anxiety symptoms among university faculty, with this association potentially operating through reduced emotional exhaustion. The strength of this association was contingent on the type of academic stress: challenge stressors appeared to strengthen the negative association between leisure-time PA and subsequent EE, whereas high-intensity hindrance stressors were associated with a reversal of the predictive pattern. These findings, given the observational design, are best interpreted as longitudinal predictive patterns rather than causal effects. They suggest that interventions targeting faculty well-being may benefit from combining administrative burden reduction with individual recovery support, though direct experimental verification is needed.

## 1. Introduction

University faculty worldwide have experienced mounting occupational pressure over the past two decades, driven by the global trend toward performance-based accountability and competitive evaluation systems (Shin & Jung, 2014). Systematic reviews indicate that burnout and chronic anxiety are widespread among academic staff across national contexts (Watts & Robertson, 2011). Within this global trend, pressure on Chinese university faculty has intensified particularly sharply: the “Double First-Class” initiative and reforms to academic personnel systems have produced an increasingly “performativity-oriented” academic labor market (Andersen et al., 2025; Y. Zhang & Tsang, 2021), where the “up-or-out” tenure-track system and competition for research grants, high-impact publications, and academic promotions generate chronic high-stress conditions (Gong & Li, 2025; Yan & Yan, 2023). Recent qualitative evidence characterizes faculty experience under these conditions as an “involution” (nei juan) spiral, in which escalating effort yields diminishing returns and sustained psychological strain (Y. L. Zhang et al., 2024). Empirical studies further document elevated occupational stress among Chinese faculty (Sun et al., 2011), with emotional exhaustion and anxiety emerging as recurrent threats to well-being and academic productivity (Jiang et al., 2017; Yang et al., 2019).

Among behavioral interventions, leisure-time physical activity has received sustained attention as an accessible, low-cost approach to occupational recovery (Wendel-Vos et al., 2004), with meta-analytic evidence supporting its anxiolytic and antidepressant effects across populations (Heissel et al., 2023; Schuch et al., 2016) and studies targeting educational workers suggesting that aerobic activity can alleviate chronic anxiety among teachers (Xu et al., 2023). However, direct longitudinal evidence on the associations linking physical activity to anxiety among university faculty remains limited, and the conditions under which this relationship holds or breaks down are not well understood.

To clarify when and how physical activity is longitudinally associated with faculty anxiety symptoms, the present study integrates three theoretical perspectives operating at complementary explanatory levels. Conservation of Resources (COR) theory specifies why chronic strain accumulates: faculty are motivated to protect valued resources, and sustained occupational demands that exceed available resources produce emotional exhaustion and anticipatory anxiety (Hobfoll et al., 2018). The Effort-Recovery Model then specifies how this accumulation can be interrupted—through off-job recovery behaviors that allow psychological detachment and resource replenishment, with leisure-time physical activity being among the most empirically supported (Sonnentag & Fritz, 2007, 2015; Tromp & Blomme, 2012). Together, these two frameworks articulate a baseline ‘resource depletion–recovery’ pathway. However, this pathway is not unconditional. The Challenge–Hindrance Stressor Model specifies when recovery behaviors function effectively: challenge stressors are appraised as surmountable and growth-promoting, leaving cognitive resources available to benefit from recovery, whereas hindrance stressors are appraised as institutional obstacles that deplete control and may turn recovery efforts themselves into additional self-regulatory demands (Cavanaugh et al., 2000; Gerich & Weber, 2020; Xie & Feng, 2024). Integrating these perspectives—COR as the motivational logic, Effort-Recovery as the behavioral pathway, and Challenge–Hindrance as the appraisal-based boundary condition—we propose that leisure-time physical activity predicts lower context-specific anxiety symptoms through reduced emotional exhaustion, and that the strength, and potentially the direction, of this pathway depends on the type of academic stress faculty are currently navigating.

Emerging evidence suggests that the recovery value of physical activity is not unconditional. Gerich and Weber (2020) report that off-job activities have ambivalent effects on burnout and job satisfaction, moderated by job control and social support, and the broader stress-coping literature indicates that coping behaviors can lose restorative function—or become additional stressors—under high time pressure and limited control (Poulus et al., 2020). Consistent with this, faculty attempting to maintain regular exercise during periods of intense administrative load or evaluation pressure may experience heightened role conflict rather than relief. These observations raise the possibility that, under hindrance-type academic stress, the predictive relationship between physical activity and emotional exhaustion may be attenuated or even reversed—a pattern that cannot be captured by treating academic stress as a unidimensional construct.

**Figure 1:**
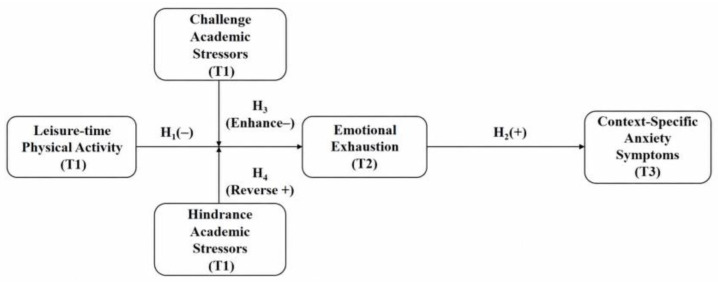
Conceptual Model of the Cross-Lagged Effects and Dual Moderating Roles.

Two gaps motivate the present study. First, most research on exercise and faculty psychological outcomes has relied on cross-sectional designs, precluding inference about temporal ordering. Second, few studies have incorporated the challenge–hindrance distinction into models of exercise-based recovery, leaving the boundary conditions of these associations underspecified. To address these gaps, we employ a three-wave cross-lagged panel design with three-month intervals among faculty from five universities in Zhengzhou, China, to examine longitudinal predictive associations among leisure-time physical activity, emotional exhaustion, and context-specific anxiety symptoms, and how these associations differ across challenge versus hindrance stressor contexts. Given the observational nature of the data, our contributions are framed as clarifying predictive patterns rather than establishing causal mechanisms. The conceptual model is presented in Figure 1.

Building on the gaps and theoretical framework outlined above, this study addresses three research questions:

**RQ1.**  *Does leisure-time physical activity longitudinally predict context-specific anxiety symptoms among university faculty over a six-month period?*

**RQ2.**  *Does emotional exhaustion mediate the longitudinal relationship between leisure-time physical activity and context-specific anxiety symptoms?*

**RQ3.**  *Do challenge and hindrance academic stressors differentially moderate the predictive relationship between leisure-time physical activity and emotional exhaustion?*

To address these research questions, we propose the following hypotheses, derived from the integrated theoretical framework outlined above:

**Hypothesis** **1 (H1).** *Leisure-time physical activity has a significant negative predictive effect on university teachers’ future emotional exhaustion.*

**Hypothesis** **2 (H2).** *Emotional exhaustion statistically mediates the longitudinal association between leisure-time physical activity and context-specific anxiety symptoms. Specifically, higher leisure-time physical activity is expected to be associated with lower subsequent context-specific anxiety symptoms through lower emotional exhaustion.*

**Hypothesis** **3 (H3).** *Challenge academic stressors moderate the negative relationship between leisure-time physical activity and emotional exhaustion, such that the negative relationship is strengthened when challenge stressors are high.*

**Hypothesis** **4 (H4).** *Hindrance academic stressors moderate the relationship between leisure-time physical activity and emotional exhaustion, such that the negative relationship is attenuated, and may even reverse, under high levels of hindrance stressors.*

## 2. Methods

### 2.1. Participants and Procedure

The current study recruited faculty members from five full-time undergraduate universities in Zhengzhou, Henan Province, China. A stratified convenience sampling method was employed to balance feasibility with disciplinary diversity. Based on institutional levels and research evaluation standards, the sampled universities included one “Double First-Class” university, two provincial key universities, and two ordinary teaching-oriented universities. The sampling process strictly controlled for the proportional distribution of academic disciplines (STEM, agriculture and medicine, humanities, and social sciences) and employment contract types (tenure-track, traditional employment, and tenured).

Participants were recruited through announcements distributed by Human Resources Offices at the five universities. Before completing the T1 questionnaire, all participants reviewed an online consent page describing the study purpose, voluntary participation, withdrawal rights, and confidentiality, and proceeded only after providing explicit consent. To enable longitudinal matching without collecting identifying information, participants generated a personal code consisting of the last four digits of a chosen phone number and the last two digits of their birth year, used solely for wave-to-wave matching. The study was approved by the Zhengzhou University Institutional Review Board (No. ZZUIRB 2025-28).

To examine the temporal ordering and longitudinal associations among variables and address common method variance (CMV), a 3-wave cross-lagged panel model (CLPM) design was utilized (Table 1). All five core variables (leisure-time physical activity, challenge academic stressors, hindrance academic stressors, emotional exhaustion, and context-specific anxiety symptoms) were measured comprehensively across all three time points. The data collection spanned six months, with a three-month interval between each wave:

Time 1 (T1, May 2025): baseline measurement during the mid-semester. Questionnaires were distributed both online and offline, yielding 600 responses. After excluding invalid responses (e.g., failing attention check items or showing obvious response patterns), 542 valid questionnaires were retained.

Time 2 (T2, August 2025): the first follow-up during the summer research-intensive period. Using a self-generated identification code (the last four digits of the participant’s phone number combined with their birth year), T1 respondents were successfully tracked, yielding 468 valid responses.

Time 3 (T3, November 2025): the second follow-up prior to the year-end performance appraisal. Ultimately, 356 valid responses were successfully matched across all three time points, resulting in an effective retention rate of 65.68%.

To rigorously evaluate the potential impact of sample attrition (*n* = 186, comprising participants who dropped out at T2 or T3) and rule out systematic attrition bias, we conducted comprehensive analyses comparing the retained sample (*n* = 356) with the dropout sample using baseline (T1) data. Chi-square (χ^2^) tests of independence revealed no significant differences between the two groups regarding demographic and occupational distributions, including gender (χ^2^(1) = 0.22, *p* = 0.639), academic title (χ^2^(2) = 0.44, *p* = 0.804), and contract type (χ^2^(2) = 0.16, *p* = 0.922). Independent samples t-tests further indicated that dropouts did not significantly differ from retained participants in T1 leisure-time physical activity (M_retained_ = 39.85, SD = 15.09 vs. M_dropout_ = 38.50, SD = 14.80; t(540) = 1.00, *p* = 0.320), T1 emotional exhaustion (M_retained_ = 3.13, SD = 0.69 vs. M_dropout_ = 3.20, SD = 0.72; t(540) = −1.10, *p* = 0.270), or T1 context-specific anxiety symptoms (M_retained_ = 3.09, SD = 0.68 vs. M_dropout_ = 3.15, SD = 0.70; t(540) = −0.97, *p* = 0.335). Although these tests do not preclude selective attrition on unmeasured variables, they suggest that the final sample did not systematically differ from dropouts on measured baseline characteristics. Missing data in subsequent analyses were handled using full information maximum likelihood (FIML) estimation.

### 2.2. Measures

All English-language scales underwent standard translation and back-translation following Brislin (1970): two bilingual researchers independently translated the items into Chinese, a third bilingual expert back-translated them, and discrepancies were resolved by panel discussion. The Chinese versions were pilot-tested with 32 faculty members from a non-participating university. Except for leisure-time physical activity, all items were rated on a 5-point Likert scale ranging from 1 (strongly disagree) to 5 (strongly agree).

#### 2.2.1. Leisure-Time Physical Activity

Physical activity was assessed using the Physical Activity Rating Scale-3 (PARS-3), a brief three-item measure translated and revised for Chinese populations by Liang (1994). The PARS-3 assesses leisure-time physical activity during the past month across three dimensions: intensity, duration per session, and frequency. Following the standard scoring procedure, the total score was calculated as Intensity × (Duration − 1) × Frequency, yielding a possible score range of 0 to 100, with higher scores indicating greater physical activity volume. Scores of ≤19, 20–42, and ≥43 indicate low, moderate, and high physical activity levels, respectively. The PARS-3 has been widely used in Chinese occupational, student, and educational samples, and recent studies have continued to support its applicability in Chinese populations (Liang, 1994; Xu et al., 2023).

#### 2.2.2. Challenge and Hindrance Academic Stressors

Academic stressors were measured using the bi-dimensional stressor scale developed by Cavanaugh et al. (2000), adapted to the higher education context. The challenge stressor dimension (6 items) measures job demands that promote personal growth and achievement (e.g., “The research projects I am currently undertaking must be completed under extremely tight deadlines”). The Cronbach’s alphas for T1, T2, and T3 were 0.88, 0.87, and 0.89, respectively. The hindrance stressor dimension (5 items) measures demands that constrain personal development and trigger job insecurity (e.g., “Cumbersome administrative forms and reimbursements take up a lot of my research time”). The Cronbach’s α values for T1, T2, and T3 were 0.91, 0.90, and 0.92, respectively.

#### 2.2.3. Emotional Exhaustion

Emotional exhaustion was assessed using the core subscale of the Maslach Burnout Inventory—General Survey (MBI-GS) (Schutte et al., 2000). This 5-item scale measures the depletion of emotional and cognitive resources due to high-intensity academic work (e.g., “I feel emotionally drained from my work at the university”). The Cronbach’s α values for T1, T2, and T3 were 0.89, 0.91, and 0.90, respectively.

#### 2.2.4. Context-Specific Anxiety Symptoms

In the present study, “context-specific anxiety symptoms” refers to context-specific, time-bounded anxiety symptoms evoked by the academic evaluation environment, rather than momentary anxiety in the strict Spielberger sense (Elwood et al., 2012); the adapted GAD-7 short form was selected to provide a brief, well-validated assessment suitable for a three-wave design. Context-specific anxiety symptoms were measured using the 4-item short form of the Generalized Anxiety Disorder scale (GAD-7) (Spitzer et al., 2006). To fit the research context, the instructions were specifically framed as “During the past month regarding your work appraisals and research progress.” A sample item is, “I have been worrying too much about future performance appraisals or academic development.” The Cronbach’s α values for T1, T2, and T3 were 0.92, 0.93, and 0.91, respectively.

#### 2.2.5. Control Variables

To rule out potential confounding effects, participants’ gender (dummy coded: 0 = female, 1 = male), age, teaching experience, academic discipline, and contract type were included in the analytical model as time-invariant covariates.

### 2.3. Data Analysis Strategy

Data processing and analyses were conducted using SPSS 27.0 and Mplus 8.3. First, Harman’s single-factor test and Confirmatory Factor Analysis (CFA) via Mplus were utilized to assess CMV, construct validity, and longitudinal measurement invariance across the three time points. Second, Mplus 8.3 was employed to construct the cross-lagged panel model (CLPM). While controlling for synchronous covariances and autoregressive paths (e.g., T1 emotional exhaustion to T2 emotional exhaustion), the significance of cross-lagged paths was estimated to establish the directional temporal associations among variables. Finally, T1 moderators (challenge and hindrance stressors) were introduced into the CLPM framework. A bootstrapping procedure with 5000 resamples was applied to calculate and test the significance of the moderated longitudinal effects on the front-stage path (Physical Activity → Emotional Exhaustion) at the 95% confidence interval (Table 1). We employed a traditional cross-lagged panel model (CLPM) rather than a random intercept CLPM (Hamaker et al., 2015) because the three-wave design is at the lower bound for reliable separation of within- and between-person variance, and the traditional CLPM provides direct comparability with the existing occupational stress literature. This limitation is addressed in Section 4.5. Interaction terms were computed as mean-centered product terms (Dawson, 2014) between the observed T1 physical activity score and the T1 stressor variables, and entered into the CLPM as additional predictors of T2 emotional exhaustion.

## 3. Results

### 3.1. Common Method Bias Testing

Although the current study employed a three-wave cross-lagged panel design (CLPM) with three-month intervals to establish temporal and psychological separation among the core variables, all data were collected via self-reported measures from the faculty members. Consequently, it was imperative to rigorously assess the potential threat of common method variance (CMV) to the validity of the findings.

To address this, we first conducted Harman’s single-factor test, a widely recognized post hoc statistical procedure. Using SPSS 27.0, all measurement items encompassing the core variables (leisure-time physical activity, challenge academic stressors, hindrance academic stressors, emotional exhaustion, and context-specific anxiety symptoms) across T1, T2, and T3 were subjected to an unrotated principal component analysis (PCA). The results revealed that, without constraining the number of factors to be extracted, 11 factors emerged with eigenvalues greater than 1.0. Notably, the first principal component accounted for only 23.41% of the total variance, which is substantially below the widely accepted empirical threshold of 40%.

To provide a more robust assessment, we further constructed a global competing model that incorporated an Unmeasured Latent Method Construct (ULMC) using Mplus 8.3. The results demonstrated that the inclusion of the common method factor did not yield a significant improvement in the overall model fit (ΔCFI < 0.01, ΔRMSEA < 0.01). Although Harman’s single-factor test has known diagnostic limitations (Podsakoff et al., 2003), these dual diagnostic procedures suggest that common method bias may not pose a severe threat to the current study, though self-report limitations should be interpreted with caution in subsequent analyses.

### 3.2. Descriptive Statistics and Correlation Analysis

Table 2 presents the means (M), standard deviations (SD), and Pearson correlation coefficients for all core variables across the three measurement points (T1, T2, and T3). The overall data distribution characteristics and bivariate correlations aligned well with our theoretical expectations, providing a solid statistical foundation for the subsequent cross-lagged panel modeling (CLPM).

Descriptive statistics revealed that at baseline (T1), university faculty members’ emotional exhaustion (M = 3.28, SD = 0.72) and context-specific anxiety symptoms (M = 3.15, SD = 0.68) were at moderately high levels and remained elevated during T2 and T3. This objectively reflects the realistic predicament of resource depletion and intense psychological strain prevalent among the faculty cohort under the current “up-or-out” tenure systems and rigorous research evaluation metrics. Furthermore, the volume of leisure-time physical activity (M = 41.25, SD = 14.86) was moderate but exhibited considerable individual variance, offering sufficient data variability to examine the behavioral intervention effects.

The longitudinal correlation analysis preliminarily uncovered the temporal evolutionary trends among the core variables:

Main effect and mediation trends: T1 leisure-time physical activity was significantly and negatively correlated with T2 emotional exhaustion (r = −0.26, *p* < 0.01) and T3 context-specific anxiety symptoms (r = −0.21, *p* < 0.01). Concurrently, T2 emotional exhaustion was significantly and positively correlated with T3 context-specific anxiety symptoms (r = 0.44, *p* < 0.01). These cross-wave bivariate associations provided preliminary support for the hypothesized longitudinal indirect association of “physical activity → emotional exhaustion → context-specific anxiety symptoms” (H1 and H2).

Temporal stability (autoregressive effects): The same constructs exhibited strong and significant positive correlations across T1, T2, and T3. For instance, the cross-wave autoregressive correlation coefficients ranged from 0.58 to 0.65 (*p* < 0.01) for emotional exhaustion and from 0.55 to 0.62 (*p* < 0.01) for context-specific anxiety symptoms. Such high temporal stability further substantiates the chronic and persistent nature of psychological stress among faculty members. It also underscores the methodological necessity of rigorously controlling for baseline autoregressive paths in the structural equation model to accurately isolate the cross-lagged net effects.

Associations with moderators: Both challenge and hindrance academic stressors were significantly and positively associated with emotional exhaustion and context-specific anxiety symptoms across all time points (*p* < 0.01), indicating their commonality as occupational stressors. However, their correlations with leisure-time physical activity were relatively weak and largely non-significant. This finding preliminarily indicates the absence of severe multicollinearity between the independent variable and the moderators, establishing a robust statistical premise for estimating the moderating effects of the interaction terms.

### 3.3. Longitudinal Measurement Invariance

In longitudinal tracking research, ensuring that latent variables possess constant measurement properties and construct meanings across different measurement points is a prerequisite for conducting cross-lagged panel analyses. If respondents’ comprehension of questionnaire items systematically shifts over time, the longitudinal estimates in subsequent models may be confounded by methodological artifacts. Therefore, this study employed Mplus 8.3 to independently test the longitudinal measurement invariance of the multi-item latent variables (challenge academic stressors, hindrance academic stressors, emotional exhaustion, and context-specific anxiety symptoms). Given that “leisure-time physical activity” was calculated as a continuous observed variable in this study, it was excluded from this latent variable invariance testing.

For each of the four latent variables, we sequentially constructed and compared three nested structural equation models:

Configural invariance model (M1): This model only required the factor structure to remain identical across T1, T2, and T3, without imposing cross-time equality constraints on parameters, serving as the baseline model. Additionally, the model allowed the measurement residuals of the homologous items to correlate over time (autocorrelated residuals) to control for item-specific systematic errors.

Metric invariance model (M2): Building upon M1, the factor loadings of the measurement items were constrained to be equal across the three time points to test the longitudinal consistency of the measurement scale.

Scalar invariance model (M3): Building upon M2, the item intercepts were further constrained to be equal across time to ensure the comparability of the latent variable means over time.

Regarding the evaluation criteria for model comparison, given the sensitivity of the chi-square (χ^2^) statistic to large sample sizes, this study adopted the recommendations of Cheung and Rensvold (2002), utilizing the change in fit indices (Δ) as the primary diagnostic criteria. A more restrictive invariance assumption is supported when ΔCFI ≥ −0.010 and ΔRMSEA ≤ 0.015.

The nested model comparison results for each latent variable are presented in Table 3. The analyses indicated the following: First, the configural invariance models (M1) for all four latent variables demonstrated good goodness-of-fit (CFIs ranging from 0.960 to 0.968, RMSEAs < 0.060), confirming the stability of the factor structures across time. Second, in the metric invariance models (M2) with constrained factor loadings, the decreases in CFI compared to M1 ranged from 0.001 to 0.002, and the ΔRMSEA values were also within the critical thresholds, indicating longitudinal consistency in the measurement units. Finally, in the scalar invariance tests (M3), compared to M2, the changes in fit indices for each variable remained within acceptable limits (maximum ΔCFI drop of −0.003, maximum ΔRMSEA change of 0.000), thus passing the intercept invariance tests.

In conclusion, the core measurement instruments employed in this study exhibited robust scalar measurement invariance over the longitudinal tracking period. Faculty members’ cognitive baselines regarding the relevant constructs did not systematically shift over time, which provides a psychometric foundation for the estimation of subsequent cross-lagged longitudinal effects.

### 3.4. Cross-Lagged Panel Model and Longitudinal Mediation Effects

Following the establishment of longitudinal measurement invariance, a full-panel cross-lagged panel model (CLPM) was constructed using Mplus 8.3 to examine the temporal sequences and longitudinal associations among the variables. The model simultaneously estimated synchronous covariances between variables at the same measurement wave and rigorously controlled for the potential confounding effects of time-invariant covariates (i.e., gender, age, academic title, contract type, and academic discipline) on the dependent variables.

**Figure 2:**
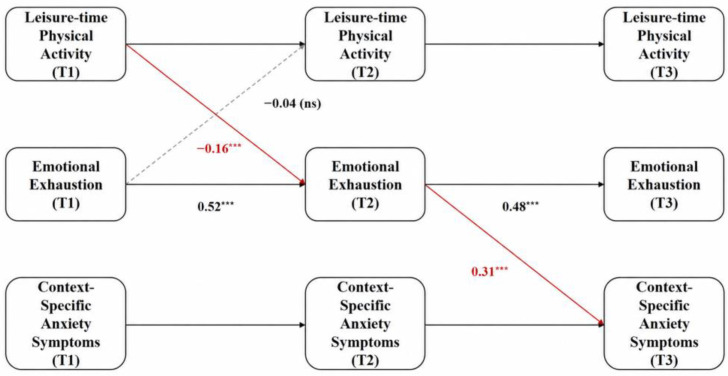
Cross-Lagged Panel Model of Leisure-time Physical Activity, Emotional Exhaustion, and Context-Specific Anxiety Symptoms. Note. ns = not significant; *** *p* < 0.001.

The global model fit indices indicated that the CLPM yielded a good fit to the empirical data: χ^2^ = 412.35, df = 215, χ^2^/df = 1.92, CFI = 0.945, TLI = 0.938, RMSEA = 0.051, SRMR = 0.046. The core path coefficients (standardized estimates, β) are detailed in Figure 2 and Table 4.

#### 3.4.1. Autoregressive and Cross-Lagged Main Effects (H1)

The path analysis results demonstrated that all cross-wave autoregressive paths for the core variables reached statistical significance. For instance, T1 emotional exhaustion significantly and positively predicted T2 emotional exhaustion (β = 0.52, *p* < 0.001), and T2 emotional exhaustion significantly and positively predicted T3 emotional exhaustion (β = 0.48, *p* < 0.001). This indicates substantial temporal continuity in university faculty’s stress responses and emotional states.

Controlling for these robust autoregressive effects, the cross-lagged path analysis revealed that T1 leisure-time physical activity had a significant negative predictive effect on T2 emotional exhaustion (β = −0.16, *p* < 0.01). Conversely, the reverse cross-lagged path—specified as a competing temporal association within the model—estimating the predictive association from T1 emotional exhaustion to T2 leisure-time physical activity was not significant (β = −0.04, *p* > 0.05). This result is consistent with a directional temporal association wherein physical activity longitudinally predicts lower subsequent emotional exhaustion, though causal inference remains limited by the observational design. Therefore, Hypothesis 1 (H1) was supported by the empirical data.

#### 3.4.2. Longitudinal Mediation Effect (H2)

In the subsequent structural path, T2 emotional exhaustion exhibited a significant positive predictive effect on T3 context-specific anxiety symptoms (β = 0.31, *p* < 0.001). To evaluate the longitudinal indirect pathway linking T1 leisure-time physical activity to T3 context-specific anxiety symptoms through T2 emotional exhaustion, this study employed a bias-corrected non-parametric percentile Bootstrap method with 5000 resamples to estimate the confidence interval of the indirect effect. The analysis revealed that the standardized estimate of this longitudinal indirect effect was −0.050, with a 95% Confidence Interval (CI) of [−0.088, −0.017]. Because this confidence interval does not contain 0, the indirect effect is statistically significant at the 0.05 level. Consequently, emotional exhaustion statistically mediated the longitudinal association between early leisure-time physical activity and subsequent context-specific anxiety symptoms among university faculty. Thus, Hypothesis 2 (H2) was supported.

### 3.5. Longitudinal Moderation Effects

To examine the longitudinal moderating effects of the two distinct types of academic stressors (challenge and hindrance academic stressors) on the relationship between leisure-time physical activity and emotional exhaustion, this study further introduced observed mean-centered product terms based on the full cross-lagged panel model.

Prior to the moderation analysis, to reduce the potential interference of multicollinearity on parameter estimation, the independent variables (T1 leisure-time physical activity) and the moderating variables (T1 challenge academic stressors and T1 hindrance academic stressors) were mean-centered. Subsequently, two interaction terms, “T1 Leisure-time Physical Activity × T1 Challenge Academic Stressors” and “T1 Leisure-time Physical Activity × T1 Hindrance Academic Stressors,” were constructed to examine their predictive effects on T2 emotional exhaustion. In these equations, T1 emotional exhaustion and all demographic covariates remained strictly controlled.

#### 3.5.1. The Moderating Effect of Challenge Academic Stressors (H3)

**Figure 3:**
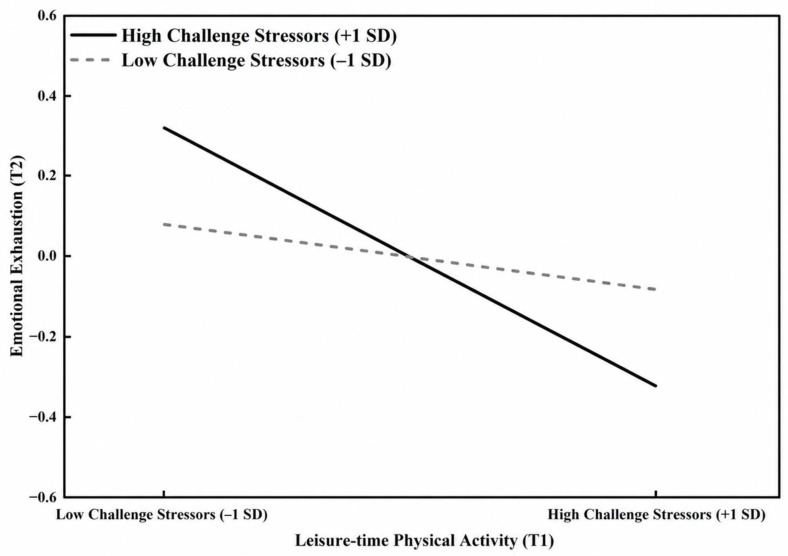
Moderating Effect of Challenge Academic Stressors on the Relationship between Leisure-time Physical Activity and Emotional Exhaustion.

The data analysis results indicated that the interaction term “T1 Leisure-time Physical Activity × T1 Challenge Academic Stressors” had a significant negative predictive effect on T2 emotional exhaustion (β = −0.15, *p* < 0.05). To further reveal the specific pattern of this moderating effect, a simple slope analysis was conducted at one standard deviation above (+1 SD) and below (−1 SD) the mean of challenge academic stressors, and an interaction plot was generated (Figure 3). The simple slope test revealed that when challenge academic stressors were at a high level (+1 SD), the negative predictive effect of T1 leisure-time physical activity on T2 emotional exhaustion was more significant with a larger effect size (Simple Slope = −0.32, t = −3.85, *p* < 0.001). Conversely, when challenge academic stressors were at a low level (−1 SD), this negative predictive effect was relatively weak and did not reach statistical significance (Simple Slope = −0.08, t = −1.82, *p* > 0.05). This result suggests that challenge stressors may strengthen the association between physical activity and lower emotional exhaustion. Thus, Hypothesis 3 (H3) was supported by the empirical data.

#### 3.5.2. The Moderating Effect of Hindrance Academic Stressors (H4)

**Figure 4:**
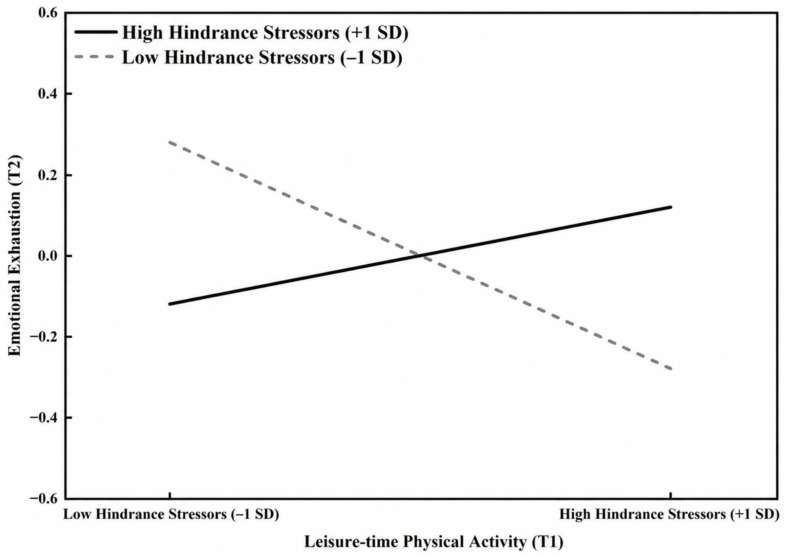
Moderating Effect of Hindrance Academic Stressors on the Relationship between Leisure-time Physical Activity and Emotional Exhaustion.

In contrast to the above findings, the interaction term “T1 Leisure-time Physical Activity × T1 Hindrance Academic Stressors” exhibited a significant positive predictive effect on T2 emotional exhaustion (β = 0.22, *p* < 0.001). The simple slope analysis (Figure 4) further clarified the pattern of this interaction: under conditions of low hindrance academic stressors (−1 SD), T1 leisure-time physical activity significantly and negatively predicted T2 emotional exhaustion (Simple Slope = −0.28, t = −3.52, *p* < 0.001). However, under high levels of hindrance academic stressors (+1 SD), this negative association was no longer observed; instead, the slope reversed and became significantly positive for T2 emotional exhaustion (Simple Slope = 0.12, t = 2.14, *p* < 0.05). This statistical outcome indicates that under highly depleting hindrance stressors, increasing leisure-time physical activity not only failed to predict lower resource depletion but was associated with higher subsequent emotional exhaustion among university faculty in the present sample. Therefore, Hypothesis 4 (H4) was supported, though the reversal effect warrants cautious interpretation.

## 4. Discussion

Employing a three-wave cross-lagged panel design spanning six months, this study examined longitudinal predictive associations among leisure-time physical activity, emotional exhaustion, and context-specific anxiety symptoms among university faculty. It also examined whether challenge and hindrance academic stressors moderated the association between leisure-time physical activity and subsequent emotional exhaustion. Overall, the findings suggest that leisure-time physical activity was associated with lower subsequent emotional exhaustion under some stressor conditions, whereas this association was attenuated or reversed under high hindrance stress. This section discusses these findings in conjunction with the empirical data and the observational nature of the design.

### 4.1. Main Effects and Longitudinal Mediation Mechanisms

The results of the cross-lagged model revealed that, after controlling for baseline autoregressive effects, T1 leisure-time physical activity was a significant negative predictor of T2 emotional exhaustion (β = −0.16, *p* < 0.01), which in turn was positively associated with T3 context-specific anxiety symptoms. Bootstrap tests supported a longitudinal indirect predictive pathway from leisure-time physical activity to context-specific anxiety symptoms via emotional exhaustion (95% CI [−0.088, −0.017]). We use the language of prediction rather than causation throughout the Discussion to reflect the observational nature of the design.

This pattern is broadly consistent with the meta-analytic findings of Heissel et al. (2023) and Schuch et al. (2016) on the anxiolytic effects of exercise. At the same time, it is important to note that not all studies converge on a uniformly positive picture. Gerich and Weber (2020) reported that the effects of off-job activities on burnout and job satisfaction are ambivalent and contingent on job control and social support, and Guo (2025) similarly showed that the mental-health benefits of exercise operate through intervening psychological processes rather than as a direct effect. Our results align with this more conditional view: physical activity predicts lower exhaustion on average, but the effect is neither universal nor mechanistically simple.

We interpret the mediation pathway in light of the Effort-Recovery Model (Tromp & Blomme, 2012) and the psychological detachment framework (Sonnentag & Fritz, 2007). Under the current academic evaluation system, university faculty chronically face high workloads (Sun et al., 2011), and sustained effort investment without adequate recovery is likely to accumulate in chronic emotional exhaustion. Regular leisure-time physical activity functions as an active recovery strategy that encourages attentional disengagement from work demands and facilitates psychological detachment. The exercise process may also support recovery through physiological mechanisms and enhanced self-efficacy. Without such recovery, initial exhaustion may solidify over time and eventually manifest as anticipatory anxiety regarding career prospects, which is particularly salient among faculty operating under “up-or-out” tenure pressure (Gong & Li, 2025).

It should be emphasized, however, that we did not directly measure the proposed recovery processes themselves—psychological detachment, recovery experiences, and resource replenishment were not assessed as intermediate variables in our design. Emotional exhaustion in our model functions as a statistical mediator rather than as a direct indicator of recovery. The Effort-Recovery framework therefore provides a theoretical interpretation of the observed longitudinal pattern, not a tested mechanism. Future research incorporating direct measures of recovery experiences, psychological detachment, and sleep quality would help disentangle whether the predictive effect of physical activity on exhaustion primarily operates through resource recovery, or through alternative pathways such as distraction, rumination reduction, or physiological arousal regulation. Unmeasured third variables—including workload fluctuations, sleep, personality, and institutional climate—may also jointly influence physical activity and emotional exhaustion, and the present observational design cannot rule out their contribution to the observed associations.

Two further interpretive cautions are warranted. First, the longitudinal predictive direction we observed does not preclude reverse causal processes. Faculty experiencing higher emotional exhaustion may turn to physical activity as a form of compensatory coping, such that the negative cross-lagged path between PA and EE could partially reflect a “self-medication” pattern rather than (or in addition to) a recovery effect. Although our reverse cross-lagged paths from T1 EE to T2 PA were not statistically significant, non-significance does not establish unidirectionality, particularly given the modest effect sizes observed. Second, our findings are not uniformly consistent with the broader literature on exercise and occupational well-being. While meta-analyses generally support the protective role of physical activity (Heissel et al., 2023; Schuch et al., 2016), several rigorous longitudinal studies have reported null or even adverse associations under specific occupational conditions. Häusser and Mojzisch (2017), for instance, found that the recovery benefits of off-job activities are heavily moderated by job control, and that physical activity itself may fail to produce restorative effects when undertaken in chronically depleted states. The present results are best understood as one observation in this larger and still-unsettled empirical landscape, rather than as definitive evidence of a protective pathway.

### 4.2. The Dual Moderating Effects of Academic Stressors

A second finding concerns the contrasting moderating roles of challenge and hindrance academic stressors in the relationship between leisure-time physical activity and emotional exhaustion.

Under high challenge academic stressors, the negative predictive effect of physical activity on T2 emotional exhaustion was stronger (Simple Slope = −0.32, *p* < 0.001). Drawing on the Challenge–Hindrance Stressor Model (Cavanaugh et al., 2000), challenge stressors such as competitive grant applications and core curriculum development, although demanding, are typically appraised as surmountable and as offering potential gains in competence and career advancement. In such appraisal contexts, physical activity may provide physiological and psychological resources that complement the motivational activation elicited by challenge demands (Fletcher & Sarkar, 2013). This is consistent with, but does not directly test, a resource-synergy interpretation.

Under high hindrance academic stressors, the pattern was reversed: higher T1 physical activity was associated with higher T2 emotional exhaustion (Simple Slope = 0.12, *p* < 0.05). Hindrance stressors such as cumbersome administrative procedures, opaque evaluation metrics, and unproductive meetings are generally appraised as institutional obstacles that impede goal attainment and generate powerlessness and time pressure (Poulus et al., 2020). One plausible interpretation is that, for faculty already experiencing severe time scarcity and depletion, sustaining a regular exercise routine becomes an additional self-regulatory demand rather than a restorative activity, intensifying role conflict between “exercise time” and “catching up on delayed work.”

We acknowledge, however, that this interpretation is not the only one the data can support. First, individuals experiencing higher hindrance stress may already be in worse physical or psychological condition at T1, such that their exercise engagement reflects compensatory coping rather than genuine recovery. Second, unmeasured time-varying variables—workload intensity, sleep deprivation, and work–home conflict—may simultaneously increase both exercise effort and subsequent exhaustion. Third, because the moderators were measured only at T1 and treated as static, we cannot capture within-person fluctuations in challenge and hindrance stress across teaching and evaluation cycles. Temporally dynamic moderation effects therefore remain an open question.

It is also worth noting that challenge and hindrance stressors were not simply opposites in our data: both were positively associated with emotional exhaustion and anxiety in the zero-order correlations. The moderation findings should therefore be understood as revealing differential amplification or attenuation of the association between physical activity and lower emotional exhaustion, rather than implying that challenge stressors are benign. The appraisal distinction matters for how recovery behaviors function, but both stressor types still impede faculty well-being.

This differential pattern has particular significance within the Chinese higher education context. The “up-or-out” tenure-track reform and performance-based evaluation systems associated with the “Double First-Class” initiative have substantially intensified hindrance-type stressors for faculty—frequent evaluation metric changes, compressed publication timelines, and heavy administrative reporting (Andersen et al., 2025; Gong & Li, 2025; Ha & Sung, 2011). Under these institutional conditions, the kind of reversal effect observed here may be especially pronounced. Whether the same pattern generalizes to higher education systems with different evaluation cultures is an empirical question for cross-cultural replication.

Given these considerations, the observed reversal is better described as a conditional pattern warranting further investigation than as a robust “exercise paradox.” Replication in other samples, with dynamic measurement of moderators and direct assessment of recovery mechanisms, is needed before this pattern can be treated as a stable phenomenon.

Beyond the alternative explanations enumerated above, the reversal pattern under hindrance stress also resonates with two adjacent branches of the literature. The overtraining and exercise-stress literature (Kreher & Schwartz, 2012) suggests that physical activity can shift from restorative to depleting when undertaken without adequate recovery resources—a transition that may be especially likely when individuals are already depleted by hindrance-type occupational stressors. Similarly, the work–family conflict literature (Allen et al., 2000) indicates that role demands competing for finite time resources amplify rather than buffer strain, and the time investment required to maintain an exercise routine may operate as exactly such a competing demand for hindrance-burdened faculty. These adjacent frameworks do not replace our recovery-based interpretation but situate it within a broader understanding of when behavioral resources flip valence under chronic strain. We emphasize that our data do not directly test these mechanisms; they are offered to guide future research that incorporates direct measures of overtraining symptoms, role-time allocation, and work–family conflict alongside physical activity and stress assessments.

### 4.3. Theoretical Contributions

#### This Study Offers Four Theoretical Contributions

First, it extends the Challenge–Hindrance Stressor Model (Cavanaugh et al., 2000) by demonstrating that challenge and hindrance stressors not only differ in their direct effects on outcomes but also function as contextual moderators that shape the efficacy of off-job recovery behaviors. Prior work has largely focused on direct effects; our findings suggest that the appraisal context in which recovery occurs is itself a boundary condition for recovery effectiveness.

Second, it identifies a boundary condition for the Effort-Recovery Model (Tromp & Blomme, 2012). The assumption that off-job activity promotes resource replenishment may not hold uniformly across stress contexts. Under conditions of depletion combined with hindrance stress, the same behavior that typically restores resources may impose additional self-regulatory demands. This points to a more conditional version of the Effort-Recovery framework in which the recovery value of a behavior depends on the broader stress appraisal environment.

Third, by applying a three-wave longitudinal design to a sample of Chinese university faculty operating under an intensified performance-evaluation regime, the study provides context-specific evidence about how institutional pressures shape recovery processes. The findings should be understood as pertaining to this specific cultural and institutional context rather than as universal claims; their generalizability to other higher education systems requires cross-cultural replication.

Fourth, the present study contributes to an ongoing methodological dialogue in occupational health psychology regarding the interpretive limits of cross-lagged panel models for theorizing about within-person change. Our findings illustrate both the value and the constraints of three-wave traditional CLPM evidence: the data identify temporal predictive structures that cross-sectional designs cannot, while simultaneously underscoring that such structures cannot, on their own, adjudicate between “recovery” and “compensatory coping” interpretations of the same statistical pattern. This methodological tension—productive but unresolved—suggests that future theoretical work on physical activity and occupational well-being may benefit from explicitly distinguishing between-person predictive theories (which CLPM-style evidence can inform) and within-person change theories (which require RI-CLPM, intensive longitudinal designs, or experimental manipulation to test). Locating our findings within this distinction reframes the present study’s contribution as primarily speaking to the former rather than the latter.

### 4.4. Practical Implications

The findings of this study carry tentative implications for university human resource management and for individual faculty, though the observational design means that these implications require empirical verification before being translated into policy.

At the institutional level, the results point to the importance of distinguishing between stressor types when designing faculty support programs. Simply promoting faculty participation in physical activity, without attending to the broader stress environment, may be insufficient and—under high hindrance stress conditions—potentially counterproductive. Specific organizational actions that align with the present findings include: (a) streamlining administrative reporting requirements and reducing the frequency of non-essential meetings to preserve continuous time blocks for faculty work and recovery; (b) stabilizing evaluation criteria over longer cycles and communicating them transparently, so that evaluation demands function more as clear-goal challenge stressors than as opaque hindrance stressors; and (c) providing flexible on-campus exercise facilities and scheduling options that lower the time cost of physical activity. These suggestions are consistent with, but not directly tested by, the present study.

At the individual level, the findings suggest that faculty may benefit from adjusting recovery strategies according to the type of stress they are currently facing. During periods dominated by challenge demands, sustained physical activity may support performance and recovery simultaneously. During periods dominated by hindrance stress, rigidly maintaining a demanding exercise routine may add to self-regulatory load; low-intensity, low-cognitive-demand recovery activities, or social support-based coping, may be more appropriate in those windows. We emphasize that these are tentative suggestions grounded in a single observational sample and should not be interpreted as prescriptive guidelines.

### 4.5. Limitations and Future Directions

Several limitations of the present study should be acknowledged, which also point to directions for future research.

First, although the three-wave longitudinal design with three-month intervals strengthens the temporal ordering among variables, the traditional cross-lagged panel model (CLPM) conflates within-person change with stable between-person differences. The relatively strong autoregressive paths observed in our model suggest that a substantial portion of variance in the key constructs is stable, which limits the extent to which cross-lagged paths can be interpreted as evidence of within-person change processes. Furthermore, although key demographic covariates were controlled, statistical adjustment does not constitute a true counterfactual comparison, and the observational nature of the design precludes strong causal inference. Future research with four or more waves should apply the random intercept CLPM (Hamaker et al., 2015) to isolate within-person effects, and quasi-experimental or intervention-based designs are encouraged to further test the longitudinal associations observed here.

Second, all study variables were assessed via faculty self-report. Despite procedural safeguards including three-month temporal separation between measurements, varied response formats across scales, and both Harman’s single-factor and ULMC diagnostics, common method variance cannot be fully ruled out. Future research should incorporate other-rated data, objective behavioral data, or physiological indicators to reduce reliance on a single reporting source.

Third, the adapted 4-item GAD-7 short form used to assess context-specific anxiety symptoms was originally developed to capture generalized anxiety symptoms rather than context-specific anxiety symptoms in the classical Spielberger sense, which distinguishes momentary anxiety from trait anxiety (Elwood et al., 2012). Although we conceptualized the measure as capturing context-specific, time-bounded anxiety symptoms evoked by the academic evaluation environment, this work-context adaptation has not been independently psychometrically validated. Future research should develop and validate a measure specifically designed to capture work-related anxiety symptoms among university faculty.

Fourth, the present study did not incorporate several potentially relevant confounding variables that may simultaneously influence physical activity, emotional exhaustion, and anxiety, such as sleep quality, workload intensity, personality traits, prior mental health history, and institutional climate. These unmeasured factors may partially account for the observed longitudinal associations. Future research should include more comprehensive baseline and time-varying covariates to more rigorously isolate the independent effects of physical activity.

Fifth, challenge and hindrance academic stressors were measured only at T1 and treated as static moderators in our analyses. However, academic stress experienced by university faculty is likely to fluctuate meaningfully across teaching cycles and evaluation periods. Future research should consider measuring moderators at multiple waves to capture their dynamic variation and time-varying interactions with the recovery function of physical activity.

Sixth, a stratified convenience sample of faculty from five universities in Zhengzhou was used, which is not statistically representative of the broader population of Chinese university faculty. Selective participation bias is also possible, as faculty members with stronger interest in physical activity or mental health issues may have been more inclined to participate. Future research should extend to faculty across different regions, institutional types, and tiers to test the external validity of the present findings.

Seventh, the findings are embedded within the specific institutional context of Chinese higher education, including the “up-or-out” tenure pressure, performance-based evaluation culture, and the intensified research assessment regime under the “Double First-Class” initiative. These institutional arrangements profoundly shape the nature and intensity of the challenge and hindrance stressors faculty experience. The extent to which the “exercise paradox” and the differential moderating effects of challenge versus hindrance stressors generalize to higher education systems in other national and cultural contexts remains an empirical question that warrants cross-cultural replication.

Eighth, the “exercise paradox” identified in this study warrants cautious interpretation. Although the data suggest that physical activity was associated with higher emotional exhaustion under high hindrance stress, this reversal effect may also reflect unmeasured third variables or reverse causal processes. The robustness and boundary conditions of this phenomenon should be replicated in diverse samples before it is translated into organizational intervention recommendations.

Finally, beyond the methodological limitations enumerated above, the present study is constrained by what it does not measure. We do not have data on objective physical activity (e.g., wearable devices), physiological recovery indicators (e.g., cortisol, heart rate variability), or work–nonwork boundary management practices, all of which would substantially strengthen mechanistic inference. The reliance on self-reported PARS-3 scores also means that our exposure variable conflates the frequency, intensity, and subjective experience of exercise; future research disaggregating these dimensions—particularly distinguishing high-arousal performance-oriented exercise from low-arousal recovery-oriented exercise—would clarify whether the reversal pattern under hindrance stress reflects exercise type rather than exercise occurrence.

## 5. Conclusions

In this three-wave observational study, regular leisure-time physical activity was longitudinally associated with lower subsequent emotional exhaustion among university faculty, which in turn was associated with lower context-specific anxiety symptoms. The strength of this longitudinal association, however, appears to depend on the nature of academic stress: challenge academic stressors may strengthen the negative longitudinal association between physical activity and subsequent emotional exhaustion, whereas under high-intensity hindrance stressors, physical activity was associated with higher subsequent exhaustion in the present sample. Because the design is observational and based on a traditional cross-lagged panel model, these findings are best understood as longitudinal predictive patterns rather than as evidence of causal effects, and they require replication and experimental verification before being translated into intervention recommendations. With this caveat in mind, our results tentatively suggest that occupational health initiatives for university faculty may benefit from combining organizational efforts to reduce hindrance-type administrative demands with individual recovery strategies flexibly matched to the current stress context.

## Figures and Tables

**Table 1:** Demographic characteristics of participants (N = 356).

Variables	Categories	Frequency (*n*)	Percentage (%)
Gender	Male	192	53.9
	Female	164	46.1
Age	≤30 years	42	11.8
	31–45 years	258	72.5
	≥46 years	56	15.7
Academic title	Lecturer/Assistant Prof.	185	52.0
	Associate Prof.	124	34.8
	Professor	47	13.2
Contract type	Tenure-track	140	39.3
	Traditional employment	155	43.5
	Tenured & Others	61	17.2
Discipline	STEM	148	41.6
	Agriculture & Medicine	54	15.2
	Humanities & Social Sciences	154	43.2
Teaching experience	≤5 years	135	37.9
	6–15 years	160	44.9
	≥16 years	61	17.2
Total		356	100.0

**Table 2:** Means, Standard Deviations, and Pearson Correlations (N = 356).

Variables	M	SD	1	2	3	4	5	6	7	8	9	10	11	12	13	14
PA_T1	41.25	14.86	1													
CS_T1	3.52	0.62	0.04	1												
HS_T1	3.24	0.76	−0.06	0.12 *	1											
EE_T1	3.28	0.72	−0.15 **	0.28 **	0.42 **	1										
SA_T1	3.15	0.68	−0.12 *	0.24 **	0.38 **	0.52 **	1									
PA_T2	40.80	15.12	0.72 **	0.05	−0.08	−0.18 **	−0.14 **	1								
CS_T2	3.48	0.65	0.03	0.68 **	0.15 **	0.22 **	0.20 **	0.06	1							
HS_T2	3.29	0.78	−0.05	0.10	0.75 **	0.36 **	0.33 **	−0.07	0.14 **	1						
EE_T2	3.35	0.75	−0.26 **	0.25 **	0.39 **	0.65 **	0.45 **	−0.19 **	0.31 **	0.46 **	1					
SA_T2	3.22	0.70	−0.18 **	0.22 **	0.35 **	0.48 **	0.62 **	−0.15 **	0.26 **	0.40 **	0.55 **	1				
PA_T3	40.55	15.40	0.65 **	0.02	−0.07	−0.14 **	−0.11 *	0.76 **	0.04	−0.09	−0.22 **	−0.16 **	1			
CS_T3	3.50	0.63	0.05	0.58 **	0.11 *	0.20 **	0.18 **	0.04	0.71 **	0.12 *	0.25 **	0.22 **	0.05	1		
HS_T3	3.31	0.80	−0.04	0.09	0.66 **	0.32 **	0.29 **	−0.05	0.11 *	0.78 **	0.41 **	0.36 **	−0.06	0.15 **	1	
EE_T3	3.40	0.78	−0.20 **	0.21 **	0.34 **	0.56 **	0.41 **	−0.25 **	0.28 **	0.42 **	0.68 **	0.48 **	−0.21 **	0.33 **	0.48 **	1
SA_T3	3.30	0.74	−0.21 **	0.19 **	0.31 **	0.42 **	0.55 **	−0.19 **	0.24 **	0.38 **	0.44 **	0.65 **	−0.18 **	0.27 **	0.41 **	0.58 **

Note. N = 356. PA = Leisure-Time Physical Activity; CS = Challenge Academic Stressors; HS = Hindrance Academic Stressors; EE = Emotional Exhaustion; SA = Context-Specific Anxiety Symptoms. T1, T2, and T3 denote the three measurement waves. Bold values represent key cross-lagged and mediational pre-test correlation coefficients. ** *p* < 0.01, * *p* < 0.05.

**Table 3:** Longitudinal measurement invariance tests for core latent variables (N = 356).

Variables	Models	χ^2^	df	CFI	TLI	RMSEA	SRMR	Comparison	ΔCFI	ΔRMSEA
Challenge Stressors	M1: Configural	250.12	132	0.962	0.955	0.050	0.042	-	-	-
	M2: Metric	265.45	142	0.960	0.954	0.049	0.044	M2–M1	−0.002	−0.001
	M3: Scalar	288.76	152	0.957	0.953	0.050	0.045	M3–M2	−0.003	0.001
Hindrance Stressors	M1: Configural	160.54	87	0.965	0.958	0.048	0.039	-	-	-
	M2: Metric	172.31	95	0.964	0.958	0.047	0.041	M2–M1	−0.001	−0.001
	M3: Scalar	186.22	103	0.962	0.959	0.047	0.041	M3–M2	−0.002	0.000
Emotional Exhaustion	M1: Configural	175.28	87	0.960	0.952	0.053	0.043	-	-	-
	M2: Metric	188.15	95	0.958	0.953	0.052	0.045	M2–M1	−0.002	−0.001
	M3: Scalar	203.41	103	0.955	0.951	0.052	0.047	M3–M2	−0.003	0.000
Context-Specific Anxiety Symptoms	M1: Configural	105.62	51	0.968	0.959	0.055	0.035	-	-	-
	M2: Metric	114.27	57	0.966	0.960	0.053	0.038	M2–M1	−0.002	−0.002
	M3: Scalar	125.83	63	0.963	0.958	0.053	0.040	M3–M2	−0.003	0.000

Note. N = 356. M1 imposed no cross-time constraints; M2 constrained factor loadings to be equal over time; M3 constrained both factor loadings and intercepts to be equal over time. Correlated residuals over time were allowed for all homologous items across models.

**Table 4:** Standardized path coefficients of the cross-lagged panel model (N = 356).

Predictors	Dependent Variables					
	T2 PA	T2 EE	T2 SA	T3 PA	T3 EE	T3 SA
Autoregressive paths						
T1 PA	0.65 ***					
T1 EE		0.52 ***				
T1 SA			0.49 ***			
T2 PA				0.61 ***		
T2 EE					0.48 ***	
T2 SA						0.51 ***
Cross-lagged paths						
T1 PA		−0.16 **	−0.07			
T1 EE	−0.04		0.22 ***			
T1 SA	−0.06	0.18 **				
T2 PA					−0.14 **	−0.05
T2 EE				−0.05		0.31 ***
T2 SA				−0.03	0.15 *	

Note. N = 356. PA = Leisure-Time Physical Activity; EE = Emotional Exhaustion; SA = Context-Specific Anxiety Symptoms. Values are standardized path coefficients (β) after controlling for T1 demographic and occupational covariates. For conciseness, synchronous covariances and covariate paths are omitted from the table. Bold values highlight the core evolutionary paths relevant to testing Hypothesis 1 and Hypothesis 2. *** *p* < 0.001, ** *p* < 0.01, * *p* < 0.05.

## Data Availability

The datasets presented in this article are not readily available because of privacy and ethical restrictions regarding the participants’ sensitive information. Requests to access the datasets should be directed to the corresponding author.
